# The Beneficial Effect of Lomitapide on the Cardiovascular System in LDLr^−/−^ Mice with Obesity

**DOI:** 10.3390/antiox12061287

**Published:** 2023-06-16

**Authors:** Undral Munkhsaikhan, Young In Kwon, Amal M. Sahyoun, María Galán, Alexis A. Gonzalez, Karima Ait-Aissa, Ammaar H. Abidi, Adam Kassan, Modar Kassan

**Affiliations:** 1 Department of Physiology, University of Tennessee Health Science Center, Memphis, TN 38163, USA; 2 Department of Bioscience Research and General Dentistry, College of Dentistry, The University of Tennessee Health Science Center, Memphis, TN 38163, USA; 3 Department of Food Science and Agriculture Chemistry, McGill University, Montreal, QC H9X 3V9, Canada; 4 Faculty of Health Sciences, University Rey Juan Carlos, 28922 Alcorcón, Spain; 5 Centro de Investigación Biomédica en Red de Enfermedades Cardiovasculares (CIBERCV), ISCIII, 28029 Madrid, Spain; 6 Instituto de Química, Pontificia Universidad Católica de Valparaíso, Valparaíso 300, Chile; 7 College of Dental Medicine, Lincoln Memorial University, Knoxville, TN 37923, USA; 8 Department of Pharmaceutical Sciences, School of Pharmacy, West Coast University, Los Angeles, CA 91606, USA

**Keywords:** familial hypercholesteremia, HFD, lomitapide, cardiovascular function, vascular reactivity

## Abstract

Objectives: Homozygous familial hypercholesteremia (HoFH) is a rare, life-threatening metabolic disease, mainly caused by a mutation in the LDL receptor. If untreated, HoFH causes premature death from acute coronary syndrome. Lomitapide is approved by the FDA as a therapy to lower lipid levels in adult patients with HoFH. Nevertheless, the beneficial effect of lomitapide in HoFH models remains to be defined. In this study, we investigated the effect of lomitapide on cardiovascular function using LDL receptor-knockout mice (LDLr^−^/^−^). Methods: Six-week-old LDLr^−^/^−^ mice were fed a standard diet (SD) or a high-fat diet (HFD) for 12 weeks. Lomitapide (1 mg/Kg/Day) was given by oral gavage for the last 2 weeks in the HFD group. Body weight and composition, lipid profile, blood glucose, and atherosclerotic plaques were measured. Vascular reactivity and markers for endothelial function were determined in conductance arteries (thoracic aorta) and resistance arteries (mesenteric resistance arteries (MRA)). Cytokine levels were measured by using the Mesoscale discovery V-Plex assays. Results: Body weight (47.5 ± 1.5 vs. 40.3 ± 1.8 g), % of fat mass (41.6 ± 1.9% vs. 31.8 ± 1.7%), blood glucose (215.5 ± 21.9 vs. 142.3 ± 7.7 mg/dL), and lipid levels (cholesterol: 600.9 ± 23.6 vs. 451.7 ± 33.4 mg/dL; LDL/VLDL: 250.6 ± 28.9 vs. 161.1 ± 12.24 mg/dL; TG: 299.5 ± 24.1 vs. 194.1 ± 28.1 mg/dL) were significantly decreased, and the % of lean mass (56.5 ± 1.8% vs. 65.2 ± 2.1%) was significantly increased in the HFD group after lomitapide treatment. The atherosclerotic plaque area also decreased in the thoracic aorta (7.9 ± 0.5% vs. 5.7 ± 0.1%). After treatment with lomitapide, the endothelium function of the thoracic aorta (47.7 ± 6.3% vs. 80.7 ± 3.1%) and mesenteric resistance artery (66.4 ± 4.3% vs. 79.5 ± 4.6%) was improved in the group of LDLr^−^/^−^ mice on HFD. This was correlated with diminished vascular endoplasmic (ER) reticulum stress, oxidative stress, and inflammation. Conclusions: Treatment with lomitapide improves cardiovascular function and lipid profile and reduces body weight and inflammatory markers in LDLr^−^/^−^ mice on HFD.

## 1. Introduction

Homozygous familial hypercholesteremia (HoFH) is a rare autosomal dominant metabolic disorder mainly caused by mutation of the gene encoding for the low-density lipoprotein receptor (LDLr) [1,2,3,4]. LDLr is a transmembrane protein that plays an important role in balancing normal cholesterol. Because of defective LDLr, patients with HoFH exhibit higher levels of LDL-C from birth onward, exceeding approximately 400–1000 mg/dL [2,3,5,6,7]. The lifelong exposure to LDL-C in HoFH patients puts them at high risk of premature acute coronary syndrome (ACS), which develops at an early age. If untreated, this high concentration of LDL-C can lead to the rapid formation of atherosclerotic plaques [8], which is the underlying cause of myocardial infarction, stroke, and sudden cardiac death [9,10]. Unfortunately, if not treated, most patients with HoFH do not survive beyond age 30 without medical intervention [11,12], with some deaths recorded even before the age of 5 [13]. Thus, early identification of HoFH and immediate follow-up by effective treatment is essential. Unfortunately, the management of HoFH presents challenges in clinical practice. Due to limited treatment options, HoFH patients are minimally responsive to available drug therapies [14]. The current standard treatment, LDL apheresis or liver transplant, could reduce LDL-C in the plasma by 50% [15,16]. However, there is rapid re-accumulation of LDL-C in plasma; accordingly, LDL apheresis needs to be repeated every 1 or 2 weeks [15]. In clinics, LDL-C is directly involved in the development of atherosclerosis, and it is well documented in the literature that the vascular endothelium has a central role in atherogenesis. It is well documented that chronic increase in LDL-C is a key contributor to endothelial dysfunction [17,18,19,20,21] by triggering an increase in oxidative stress [18], inflammation [22,23], endoplasmic reticulum (ER) stress [24], and inhibition of endothelial nitric oxide signaling [25,26,27,28]. Therefore, reducing LDL-C is an essential part of treating and preventing various diseases, particularly in patients with HoFH.

Lomitapide is an oral inhibitor of microsome triglyceride transfer protein (MTP) approved by the Food and Drug Administration (FDA) for use in adults with HoFH. MTP, a lipid binding protein, plays an essential role in lipid metabolism, especially in ApoB lipoprotein assembly. Lomitapide directly binds MTP in the lumen of the endoplasmic reticulum (ER) thereby preventing the transfer of lipids and inhibiting the assembly of very low-density lipoprotein (VLDL) and chylomicrons. Inhibition of MTP prevents the transfer of triglycerides and phospholipids, thus reducing lipoprotein secretion and release into the systemic circulation [29]. Clinical trials showed that lomitapide markedly reduced plasma LDL-C [29], total cholesterol, and triglyceride (TG) levels [30,31,32]. A recent study found a reduction in inflammatory markers (TNFα, IL-7) in the plasma of patients treated for 6 months, suggesting that lomitapide may have an anti-inflammatory effect [33]. In our previous study, we showed that lomitapide had beneficial effects on the cardiovascular system of mice with obesity [34]. However, less is known about the effect of lomitapide on the cardiovascular system in the HoFH mouse model (LDLr^−^/^−^ mice). Thus, this research aims to assess the effect of lomitapide on HFD-induced hyperlipidemia and cardiovascular diseases (CVD) using LDLr^−^/^−^ mice on a high-fat diet (HFD).

## 2. Materials and Methods

### 2.1. Animal

In this study, we used male LDL receptor-knockout mice (LDLr^−^/^−^) that we purchased from Jackson Laboratories (stock number 002207, Jackson Labs, Bar Harbor, ME, USA). The housing of the LDLr^−^/^−^ mice and the experiments performed in this study followed the guidelines of the Institutional Animal Care and Use Facility of the University of Tennessee Health Science Center, Memphis, TN (IACUC no. 20-0193). The mice were placed under a 12 h light–dark cycle, a temperature of 21 °C, humidity of 50%, noise-free conditions, and food and water ad libitum. Six-week-old LDLr^−^/^−^ mice were fed either a standard diet (5.8% fat, 44.3% carbohydrate, 19.1% protein, Envigo cat# 7012) or a high-fat diet (HFD; 60% fat, 20% carbohydrate, 20% protein, Research Diets cat# D12492) for 12 weeks. At 16 weeks of age, HFD mice were administered lomitapide (1 mg/kg/day) by oral gavage for 2 weeks. The dose was determined based on the literature [15,35,36]. Body weight was measured weekly during the experiment period. At the end of the treatment period, lean and fat mass were assessed using an EchoMRI, Body Composition Analyzer (Echo Medical System, Houston, TX, USA). Mice were weighed, then placed in a clean plastic cylinder without sedation or anesthesia. The cylinder was loaded into a tubular EchoMRI system and scanned. After EchoMRI scan, all three groups of mice, the LDLr^−^/^−^ mice fed with a standard diet (LDLr^−^/^−^, control), the LDLr^−^/^−^ mice fed with a high-fat diet (LDLr^−^/^−^ HFD), and the LDLr^−^/^−^ mice fed with a high-fat diet and treated with lomitapide (LDLr^−^/^−^ HFD Lomi), were euthanized.

### 2.2. Tissue Collection, Plasmatic Levels of Lipids and Glucose

Mice were fasted overnight, and blood glucose level was determined in tail vein blood using the Care Touch Diabetes Testing Kit (Future Diagnostic, Joliet, IL, USA) according to the manufacturer’s protocol. Mice were then euthanized under isoflurane anesthesia. Blood samples were taken by cardiac puncture, then plasma was separated by centrifugation (2000× *g*, 15 min, 4 °C) and stored at −80 °C until further use [37]. As previously described [34], the lipid profile was assessed in plasma using the following assays: HDL and LDL/VLDL quantification kit (Sigma, Burlington, MA, USA, MAK045), Triglyceride quantification kit (Sigma, Burlington, MA, USA, MAK266), and Cholesterol quantification kit (Sigma, Burlington, MA, USA, MAK043), according to the manufacturer’s protocol. Arteries, such as mesenteric resistance arteries (MRA) and thoracic aorta, were harvested to determine protein levels by Western blot, gene expression by quantitative real-time (qRT-) PCR, and vascular reactivity using the wire myograph.

### 2.3. Oil O Red Staining

Mouse hearts were perfused in situ with phosphate-buffered saline (PBS) after blood sampling. The thoracic aorta was excised, cleaned of fat tissue, opened longitudinally, and stained with 0.5% Oil O Red (Abcam, ab150678) for 1 h according to the protocol provided [38]. Images of en-face aortic lesion areas were obtained by the camera under polarized light, and the plaque lesion area was quantified using NIH ImageJ. The whole aorta, aortic arch, and descending aortic lesion areas were calculated by dividing stained Oil O Red areas by the total area based on the literature [38,39].

### 2.4. Vascular Reactivity

Thoracic aorta and mesenteric resistance arteries (MRA) were used to evaluate the vascular reactivity as previously described [34]. Briefly, thoracic aorta and MRA were placed in a chilled Krebs solution of the following composition (in mM): NaCl, 118; KCl, 4.7; CaCl_2_, 2.5; KH_2_PO_4_, 1.2; MgSO_4_, 1.2; NaHCO_3_, 25; and glucose, 11. The pH of the solution after saturation with carbogen (95% O_2_ and 5% CO_2_) was 7.4. Thoracic aorta and MRA were cleaned of fat and connective tissue and cut into rings (2 mm in length). Thoracic aorta and MRA were carefully cleaned of fat and connective tissue, cut into rings (2 mm in length), and mounted in a small-vessel chamber myograph for the measurement of isometric tension. Thoracic aorta and MRA rings were pre-constricted with phenylephrine (PE). Endothelial function was determined by assessing the vascular relaxation of the vessels (thoracic aorta and mesenteric resistance arteries) by exposing them to a cumulative dose of an endothelium-dependent relaxant agent (acetylcholine (Ach) 10^−9^ to 10^−4^ M). Another series of experiments were carried out to determine the vascular relaxation to an endothelium-independent relaxant agent (sodium nitroprusside (SNP) 10^−9^ to 10^−4^ M).

### 2.5. Protein Expression

Thoracic aorta and mesenteric resistance artery were lysed in RIPA buffer. Total proteins were quantified using a PierceTM BCA Protein Assay Kit (Thermo Fisher, Waltham, MA, USA). Protein samples were resolved using 4–12% SDS polyacrylamide gel electrophoresis and transferred to nitrocellulose membranes. The membrane was blocked using 5% nonfat dry milk. Then, suitable primary antibodies were added and followed by secondary antibodies that were conjugated with horseradish peroxidase. Protein expression was determined as previously described [34]. Primary antibodies used in this study were as follows: markers for nitric oxide enzyme (Cell Signaling, Boston, MA, USA) (total (T) endothelial nitric oxide synthase (eNOS) (9586S, anti-rabbit) and phosphorylated (P) eNOS (9571S, anti-rabbit)), markers for endoplasmic reticulum (ER) stress (BIP (3177S, anti-rabbit) and CHOP (2895S, anti-mouse)), and markers for inflammation (P65 (8242S, anti-rabbit) and TNFα (3707S, anti-rabbit)). All primary antibodies were used at 1:1000 dilution. Secondary anti-rabbit (Abcam, Cambridge, UK, ab205718) or anti-mouse (Abcam, Cambridge, UK, ab205719) antibodies, at 1:10,000 dilution, were used for protein detection by the chemiluminescence method. We quantified the bands using Image Lab (Bio-Rad). GAPDH (Sigma, Burlington, MA, USA, G9545, dilution 1:10,000) was used as a loading control.

### 2.6. Gene Expression

Mouse thoracic aortas were harvested from all groups to determine gene expression for endoplasmic reticulum (ER) stress markers (BIP, ATF6, and ATF4), oxidative stress markers (NOX2), and inflammatory markers (P65, P50, TNFα, and VCAM-1). Gene expression was determined using real-time quantitative PCR as previously described [34]. Total RNA was isolated from 50 mg of thoracic aorta using RNeasy Micro Kit (Qiagen, Venlo, The Netherlands) as per the manufacturer’s instruction. The first strand RT-PCR was made by qScript cDNA Super Mix (Quanta Bioscience, Beverly, MA, USA). GAPDH was used as an internal control and fold changes in gene expression were measured using the ΔΔCt method. Supplementary Table S1 presents all the information related to the primers used in this study.

### 2.7. Electrochemiluminescence Multiplex Detection

The cytokines levels were measured by Mesoscale Discovery Mouse Pro-Inflammatory Panel kits (Mesoscale Diagnostics, Rockville, MD, USA). Briefly, the electrochemiluminescence used by MSD aids in multiplexing up to 10 analytes of interest. After completion of the treatment regimen, samples are transferred on a pre-coated MSD with analytes of choice, incubated for an hour followed by SULFA-TAG detection antibodies, and incubated for an additional hour. After washing, a read buffer is added, and when a voltage is applied to the plate, the chemical environment aids in photon release (i.e., light emission), which is captured by the reader instrument and quantifies the measured intensity of light emitted, which is proportional to the sample analyte present. The ordered kits have been customized to detect specific markers, such as mouse interleukin-6 (IL-6), neutrophil-activating protein 3 known as KC/GRO, and tumor necrosis factor-alpha (TNF-α). Mesoscale SQ120 and SECTOR Imager SI 2400A were used to read the plates as per the manufacturer’s instructions. A five-parameter logistic regression method was used to calculate sample concentration and standard curves.

### 2.8. Statistical Analysis

All results are expressed as means ± SEM. We used GraphPad Prism version 9 (GraphPad Software, San Diego, CA, USA) to analyze the data. The analytes for each group were compared using a one-way ANOVA with Tukey’s post hoc test and results with *p* < 0.05 are considered statistically significant.

## 3. Results

### 3.1. Treatment with Lomitapide Decreased Body Weight, Reduced Blood Glucose, and Enhanced Body Composition in LDLr^−^/^−^ Mice on HFD

**Figure 1:**
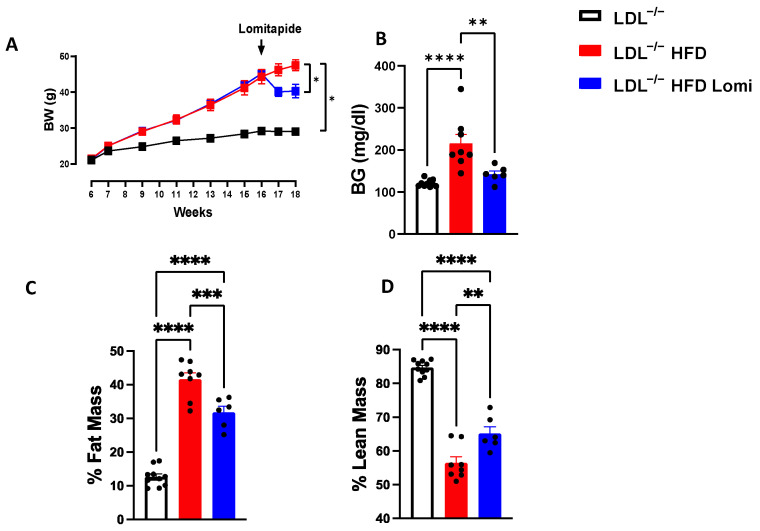
Lomitapide reduced body weight and blood glucose and ameliorated body composition profile in LDLr^−^/^−^ mice on HFD. Body weight (BW) (**A**), blood glucose (BG) (**B**), percentage of fat mass (**C**), and lean mass (**D**) in LDLr^−^/^−^ control mice and mice fed with a high-fat diet (HFD) in the presence and absence of lomitapide treatment (*n* = 6–10). * *p* < 0.05; ** *p* < 0.01; *** *p* < 0.001; **** *p* < 0.0001.

When compared to the control (LDLr^−^/^−^), mice fed with HFD (LDLr^−^/^−^ HFD) showed an increase in body weight associated with an increase in the % of fat mass (Figure 1A,C). After two weeks of lomitapide treatment, mice with HFD (LDLr^−^/^−^ HFD Lomi) displayed significantly reduced body weight (Figure 1A), which was associated with a reduction in fat mass percentage (Figure 1C). Additionally, treatment with lomitapide increased the percentage of lean mass (Figure 1D). Obesity is the most important risk factor for hyperglycemia [40]. In this study, we found that blood glucose level was significantly increased in mice fed with HFD. Treatment with lomitapide significantly reduced blood glucose as evidenced by (Figure 1B).

### 3.2. Lomitapide Treatment Decreased Lipid Profiles in LDLr^−^/^−^ Mice on HFD

**Figure 2:**
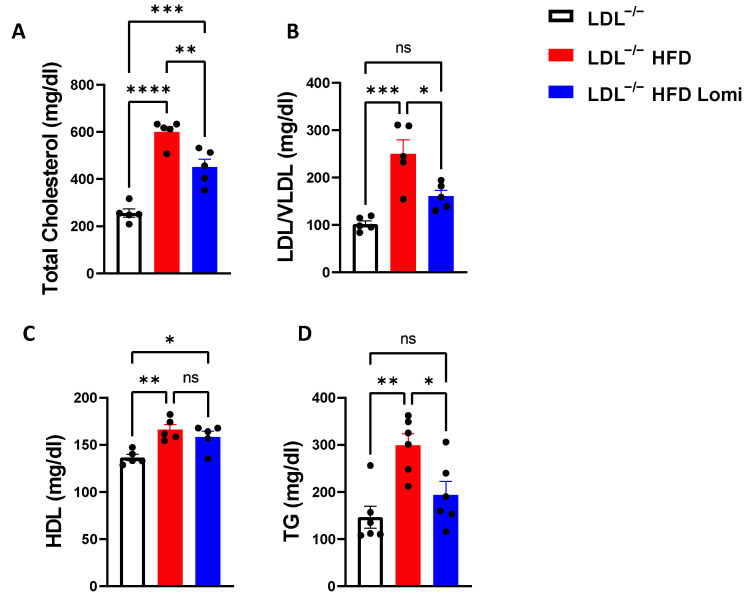
Lomitapide enhanced the lipid profile in LDLr^−^/^−^ mice on HFD. Total cholesterol (**A**), LDL/VLDL (**B**), HDL (**C**), and TG (**D**) in plasma from LDLr^−^/^−^ control mice and mice fed with a high-fat diet (HFD) treated with vehicle or lomitapide (*n* = 5–6). LDL/VLDL: low-density lipoprotein/very low-density lipoprotein; HDL: high-density lipoprotein; TG: triglyceride. ns > 0.05; * *p* < 0.05; ** *p* < 0.01; *** *p* < 0.001; **** *p* < 0.0001.

We evaluated plasma lipid profiles in all groups. Lipid levels in LDLr^−^/^−^ mice were augmented by HFD feeding when compared to lean control mice. Our data showed that total cholesterol, HDL, LDL/VLDL, and TG were higher in the HFD group compared to the control group. After treatment with lomitapide, LDL/VLDL was significantly reduced, to 161 ± 27 mg/dL, when compared to 250 ± 64 mg/dL in the HFD group. HDL levels in the HFD and lomitapide-treated groups were similar. However, the treatment was remarkably effective in reducing total cholesterol, LDL/VLDL, and TG (Figure 2).

### 3.3. Lomitapide Decreased Plaque Surface Area in the Thoracic Aorta from LDLr^−^/^−^ Mice with Obesity

**Figure 3:**
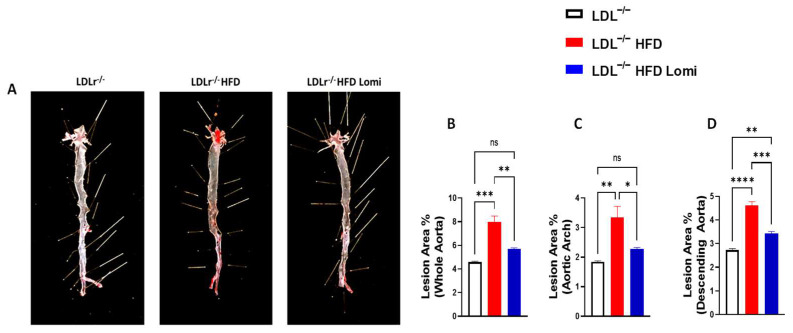
Oil O Red staining and quantification of plaque lesion area. Lomitapide decreased plaque surface area in the thoracic aorta of LDLr^−^/^−^ mice with obesity (*n* = 3). Representative images of en face staining by Oil O red of the entire aorta are shown (**A**), the whole aorta quantification of the lesion area (**B**), the aortic arch area (**C**), and the descending aorta (**D**). ns > 0.05; * *p* < 0.05; ** *p* < 0.01; *** *p* < 0.001; **** *p* < 0.0001 assessed by ANOVA followed by the Tukey test for multiple comparisons.

In Oil O red staining, the percentage of plaque area in the whole aorta in the control group (4.5 ± 0.1) was less than in the HFD group (8.0 ± 0.9). These alterations, such as lesions, were reduced by lomitapide. The percentage of the lesion area, which is the plague on the aorta in en-face view, was significantly decreased (5.7 ± 0.1) after the treatment with lomitapide shown in Figure 3.

### 3.4. Lomitapide Improved Vascular Endothelial Function in LDLr^−^/^−^ Mice on HFD

**Figure 4:**
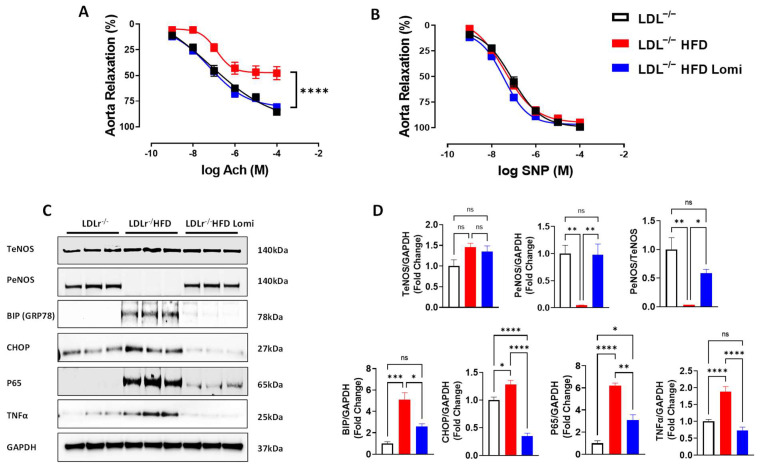
Lomitapide prevented endothelial dysfunction in the thoracic aorta from LDLr^−^/^−^ mice on HFD. Endothelium-dependent dilation to Ach (**A**), endothelium-independent dilation to SNP (**B**) (*n* = 11–14), immunoblots showing (T-eNOS, p-eNOS, BIP, CHOP, P65, TNFα, and GAPDH) (**C**), and quantification (**D**) in thoracic aorta from LDLr^−^/^−^ lean mice and LDLr^−^/^−^ obese mice treated with vehicle or lomitapide (*n* = 3–6). Ach: acetylcholine; SNP: sodium nitroprusside; T-eNOS: total endothelial nitric oxide synthase; p-eNOS: phosphorylated endothelial nitric oxide synthase; BIP: GRP78; CHOP: he C/EBP homologous protein; P65: NF-kappa-B; TNFα: tumor necrosis factor alpha. ns > 0.05; * *p* < 0.05; ** *p* < 0.01; *** *p* < 0.001; **** *p* < 0.0001.

**Figure 5:**
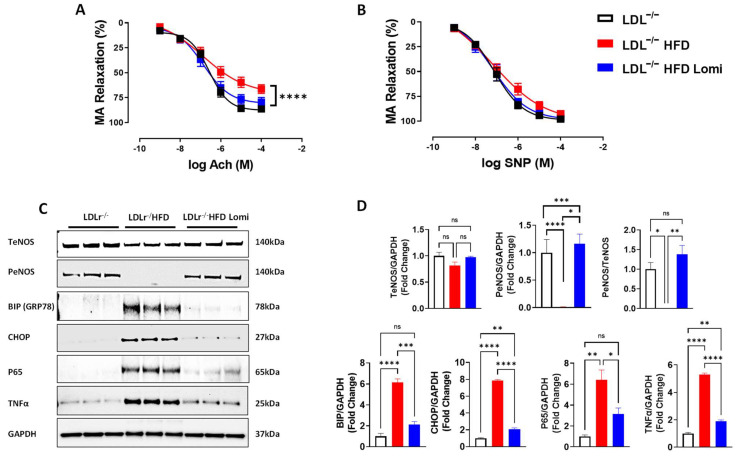
Lomitapide prevented endothelial dysfunction in MRA from LDLr^−^/^−^ mice on HFD. Endothelium-dependent dilation to Ach (**A**), endothelium-independent dilation to SNP (**B**) (*n* = 11–18), immunoblots showing (T-eNOS, p-eNOS, BIP, CHOP, P65, TNFα, and GAPDH) (**C**), and quantification (**D**) in MRA from LDLr^−^/^−^ lean mice and LDLr^−^/^−^ obese mice treated with vehicle or lomitapide (*n* = 3–6). Ach: acetylcholine; SNP: sodium nitroprusside; T-eNOS: total endothelial nitric oxide synthase; p-eNOS: phosphorylated endothelial nitric oxide synthase; BIP: GRP78; CHOP: the C/EBP homologous protein; P65: NF-kappa-B; TNFα: tumor necrosis factor alpha. ns > 0.05; * *p* < 0.05; ** *p* < 0.01; *** *p* < 0.001; **** *p* < 0.0001.

The endothelium-dependent relaxation to increasing concentrations of acetylcholine (Ach) in thoracic aortas and MRA was reduced in the HFD group compared to the control group (Figure 4A and Figure 5A). These data indicate a significant endothelial dysfunction in obese mice when compared to lean mice (Figure 4A and Figure 5A). Interestingly, treatment with lomitapide restored endothelial function in thoracic aorta (Figure 4A) and significantly improved it in MRA (Figure 5A). Endothelium-independent relaxation to sodium nitroprusside (SNP) was similar among all groups (Figure 4B and Figure 5B). It is well known that Ach induces relaxation primarily by phosphorylating the endothelial nitric oxide synthase enzyme (eNOS) [41]. In this study, we showed that PeNOS was significantly reduced in thoracic aorta and MRA from mice on HFD when compared to control lean mice (Figure 4C and Figure 5C). Interestingly, lomitapide restored the PeNOS protein level in thoracic aorta and MRA (Figure 4C,D and Figure 5C,D).

### 3.5. Lomitapide Beneficial Effect on Oxidative and Endoplasmic Reticulum Stress and Inflammation in LDLr^−^/^−^ Mice on HFD

**Figure 6:**
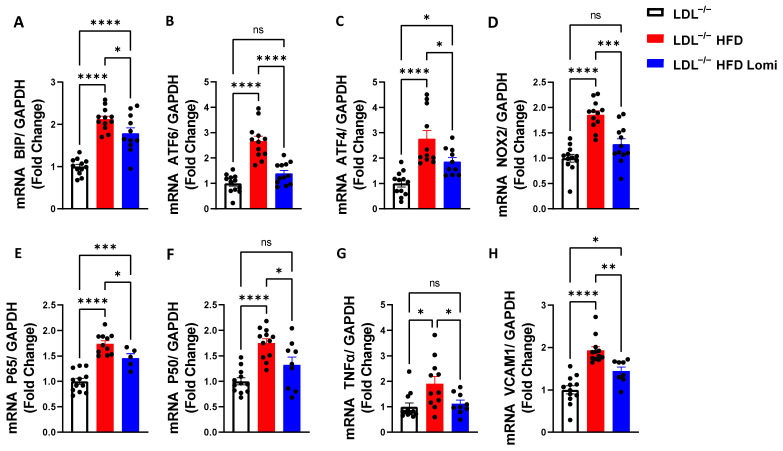
Lomitapide treatment downregulated oxidative and endoplasmic reticulum (ER) stress and inflammatory markers in thoracic aorta from LDLr^−^/^−^ mice on HFD. mRNA levels for ER stress markers (BIP, ATF6, ATF4) (**A**–**C**), oxidative stress marker (NOX2) (**D**), and inflammation markers (NFkB-p65, -p50, TNFα, VCAM1) (**E**–**H**) in thoracic aorta from LDLr^−^/^−^ lean mice and LDLr^−^/^−^ obese mice treated with vehicle or lomitapide (*n* = 8–12). BIP: GRP78; ATF6 and ATF4: activating transcription factor; p65 and p50: a subunit of NF-kappa B transcription complex; TNFα: tumor necrosis factor alpha; VCAM1: vascular cell adhesion molecule 1. ns > 0.05; * *p* < 0.05; ** *p* < 0.01; *** *p* < 0.001; **** *p* < 0.0001.

**Figure 7:**
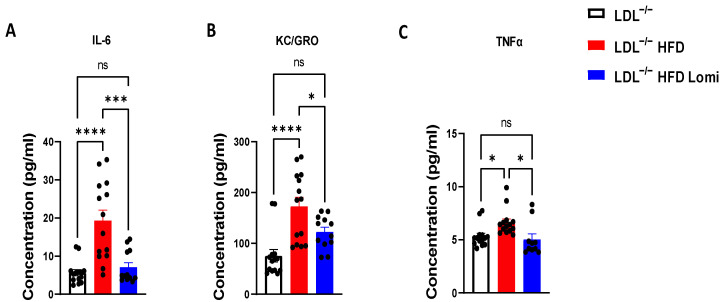
Lomitapide treatment showed an anti-inflammatory effect in LDLr^−^/^−^ mice with obesity. Lomitapide treatment significantly decreased inflammatory cytokines IL-6 (**A**), KC/GRO (**B**), and TNFα (**C**) in LDLr^−^/^−^ obese mice (*n* = 12–14). IL-6: interleukin 6; KC/GRO: neutrophil-activating protein 3; TNFα: tumor necrosis factor alpha. ns > 0.05; * *p* < 0.05; *** *p* < 0.001; **** *p* < 0.0001.

It is well known that obesity predisposes arteries to inflammation and oxidative and endoplasmic reticulum (ER) stress which eventually leads to artery damage [42]. In this study, we showed that thoracic aorta and MRA from LDLr^−^/^−^ mice on HFD display increased ER stress at both protein (BIP, CHOP; (Figure 4C,D and Figure 5C,D) and mRNA levels (BIP, ATF6, ATF4; Figure 6A–C) when compared to control lean mice. Treatment with lomitapide significantly reduced ER stress markers in both vascular beds (thoracic aorta and MRA) ((Figure 4C,D, Figure 5C,D and Figure 6A–C). Furthermore, lomitapide treatment showed a beneficial effect by decreasing the inflammation level in vessels (thoracic aorta and MRA) from mice on HFD as demonstrated by the reduction in the protein levels of TNFα and p65 (Figure 4C,D and Figure 5C,D) and mRNA levels of p65, P50, TNFα, and VCAM1 (Figure 6E–H). Endothelial dysfunction following HFD was associated with oxidative stress (Figure 6D). Lomitapide treatment significantly improved the redox system in the thoracic aorta of mice on HFD (Figure 6D). In addition, we used electrochemiluminescence via MSD V-plex to measure the level of cytokines that are involved in inflammation response and immune system regulation. We found an increased level of IL-6, KC/GRO, and TNFα in LDLr^−^/^−^ obese mice when compared to control lean mice, and treatment with lomitapide significantly decreased the cytokine levels (Figure 7).

## 4. Discussion

A previous study from our lab showed that treatment with lomitapide improved the cardiovascular system in obese mice [34]. This improvement was attributed to the rescue of the endothelium function in both conductance and resistance arteries. Owing to the novelty of these data, and lomitapide being exclusively used for homozygous familial hypercholesterolemia (HoFH) patients, we decided to investigate the effect of lomitapide on the cardiovascular system in a mouse model of HoFH (LDLr^−^/^−^ mice).

Homozygous familial hypercholesterolemia (HoFH) is a severe genetic disorder mainly caused by a mutation in the LDL receptor gene [43]. Lomitapide (MTP inhibitor) is an FDA-approved drug used as an adjunct therapy for treating adult patients with HoFH by reducing their LDL-C levels and delaying the onset of atherosclerosis [44]. Lifelong exposure to LDL-C in HoFH patients puts them at high risk of premature CVD that develops at an early age, or as a teenager, resulting in cardiovascular endothelial damage [45,46]. Surprisingly, there are no direct studies to assess the effect of lomitapide on the cardiovascular system in HoFH patients. In our study, we decided to investigate the effect of lomitapide on the cardiovascular system by using a mouse model of HoFH (LDLr^−^/^−^ mice). We induced obesity in LDLr^−^/^−^ mice by exposing them to HFD. Body weight, glucose, and lipid profile are known to be affected by HFD-induced obesity [47,48]. Our data are in agreement with these studies since we showed a steady increase in body weight coupled with perturbation in body composition evidenced by an increased % of fat mass and decreased % of lean mass in the LDLr^−^/^−^ HFD group. Interestingly, lomitapide treatment significantly reduced the body weight and the level of fat mass and increased the level of lean mass compared to the HFD group. In addition, the rise in blood glucose levels in our HFD group was significantly reduced following lomitapide treatment as well. These results indicate that lomitapide plays an important role in overturning HoFH-related complications during obesity. Our data are in agreement with a clinical study conducted on Japanese patients with HoFH. In this study, the treatment with lomitapide significantly decreased the body weight of the patients [49]. Additionally, in Zucker fatty rats, lomitapide treatment decreased their body weight and food intake, this was accompanied by a significant improvement in glucose tolerance [36]. Another study in Zucker fatty rats found that lomitapide reduced hyperphagia, prevented the increase in body weight, and improved glucose tolerance and insulin sensitivity by decreasing TG and glucose levels [50,51]. Different factors, such as the balance between energy intake and energy expenditure and physical activity, play an important role in regulating obesity and changes in body weight [52,53]. According to a clinical study conducted on Japanese patients with HoFH, body weight and serum fatty acid were reduced after lomitapide treatment without changes in energy intake [48].

Chronic elevation of LDL-C plays an important role in the development of plaques in the arteries, leading to atherosclerosis [54]. Patients with HoFH exhibit higher LDL-C levels from birth, which increases the risk of acute coronary syndrome (ACS) at an early age [5]. Clinical and preclinical studies have demonstrated that lomitapide reduces atherosclerotic risk factors by modulating postprandial lipid metabolism [55]. A study by Ueshima et al. showed that MTP inhibitors decreased atherosclerosis in apoE KO mice [56]. In the present study, the group of LDLr^−^/^−^ mice on a high-fat diet had more atherosclerotic plaque in their entire aorta compared to the control group. However, after lomitapide treatment, plaque area (%) was significantly decreased. It is well known in the literature that the greater the absolute reduction in plasma LDL-C levels, the larger the reduction in atherosclerotic cardiovascular disease risk. In a prospective clinical study, lomitapide reduced total cholesterol by 55.3%, LDL-C by 65.6%, and TG by 41.3%, and there were no significant changes in HDL [33]. In our study, the LDLr^−^/^−^ HFD group had a disturbed lipid profile when compared to control mice. Treatment with lomitapide significantly reduced total cholesterol, TG, and LDL/VLDL similar to the clinical study. Our data have solidified the concept that lomitapide reduces the progression of atherosclerosis and therefore should be considered as a potential drug for atherosclerotic cardiovascular disease.

Elevated plasma LDL-C is not only involved in the progression of atherosclerosis; it is well established that an increased level of LDL-C is a major contributor to endothelial dysfunction and its complications [19,20,21]. Increased plasma LDL-C levels contribute to the impairment of nitric oxide (NO) bioavailability due to increased production of reactive oxygen species stress [18], ER stress [24], inflammation [22,23], and alteration of the endothelial nitric oxide signaling pathways [17,27], leading to endothelial dysfunction and atherosclerosis. All these markers were increased in our obese mouse model of HoFH.

Since lomitapide was able to reduce plasma LDL-C levels, our next step was to determine if lomitapide exerts a positive effect on vascular reactivity by improving endothelial function. In the LDLr^−^/^−^ HFD group, the high level of LDL/VLDL was correlated with vascular damage evidenced by endothelial dysfunction, a renowned and recognized mechanism in obesity [21,57,58]. After lomitapide treatment, endothelial function was significantly improved. The effect of decreasing LDL/VLDL is characterized by lower oxidative and ER stress, inflammation, and improved NO activation. We also found that the pro-inflammatory cytokines, IL-6, KC/GRO, and TNFα, were decreased after lomitapide treatment in the LDLr^−^/^−^ HFD group. Collectively, our results suggest that lomitapide exerts significant therapeutic effects by decreasing endothelial dysfunction and pro-inflammatory cytokines in the HoFH mouse model.

In our recently published work, we demonstrated the positive effects of lomitapide on endothelial function in obese mice [34]. In this study, we show the positive effect of lomitapide on the cardiovascular system using the HoFH mouse model. However, lomitapide has some safety concerns. The mechanism of lomitapide involves the inhibition of VLDL and TG secretion and thus may store fat in the liver. Therefore, the side effects of lomitapide, especially liver function, raise the need to clarify this relevant concern with the future investigation.

## 5. Conclusions

**Figure 8:**
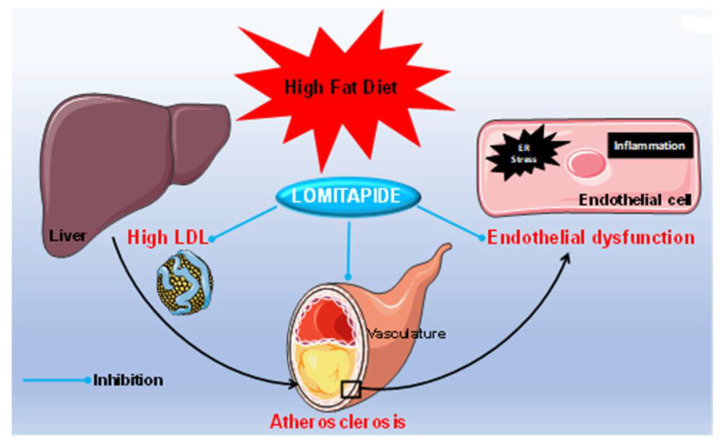
Schema summarizing the findings in this paper.

The results of our study suggest that lomitapide regulates body composition, lipid profiles, plaque area, and vascular endothelium function in both conductance (thoracic aorta) and resistance arteries (mesenteric arteries) under obese conditions. In addition, lomitapide reduces ER stress, inflammation, and oxidative stress while increasing NO activity. Therefore, lomitapide could be a potential drug to prevent or slow the progression of atherosclerosis (Figure 8).

## Data Availability

Data is contained within the article and supplementary materials.

## Supplementary Materials

The following supporting information can be downloaded at: https://www.mdpi.com/article/10.3390/antiox12061287/s1, Table S1: RT-qPCR primers are used for aorta gene expression analysis.

## References

[1] Zhang Y., Ma K.L., Ruan X.Z., Liu B.C. (2016). Dysregulation of the Low-Density Lipoprotein Receptor Pathway Is Involved in Lipid Disorder-Mediated Organ Injury. Int. J. Biol. Sci..

[2] Rader D.J., Kastelein J.J. (2014). Lomitapide and mipomersen: Two first-in-class drugs for reducing low-density lipoprotein cholesterol in patients with homozygous familial hypercholesterolemia. Circulation.

[3] Stefanutti C. (2020). Lomitapide–A Microsomal Triglyceride Transfer Protein Inhibitor for Homozygous Familial Hypercholesterolemia. Curr. Atheroscler. Rep..

[4] Pajkowski M., Dudziak M., Chlebus K., Hellmann M. (2021). Assessment of microvascular function and pharmacological regulation in genetically confirmed familial hypercholesterolemia. Microvasc. Res..

[5] Pejic R.N. (2014). Familial hypercholesterolemia. Ochsner J..

[6] Blom D.J., Averna M.R., Meagher E.A., Theron H.D.T., Sirtori C.R., Hegele R.A., Shah P.K., Gaudet D., Stefanutti C., Vigna G.B. (2017). Long-Term Efficacy and Safety of the Microsomal Triglyceride Transfer Protein Inhibitor Lomitapide in Patients With Homozygous Familial Hypercholesterolemia. Circulation.

[7] Cuchel M., Bruckert E., Ginsberg H.N., Raal F.J., Santos R.D., Hegele R.A., Wiklund O. (2014). Homozygous familial hypercholesterolaemia: New insights and guidance for clinicians to improve detection and clinical management. A position paper from the Consensus Panel on Familial Hypercholesterolaemia of the European Atherosclerosis Society. Eur. Heart J..

[8] Ison H.E., Clarke S.L., Knowles J.W., Adam M.P., Mirzaa G.M., Pagon R.A., Wallace S.E., Bean L.J., Gripp K.W., Amemiya A. (1993). Familial Hypercholesterolemia. GeneReviews^®^.

[9] Enas E.A., Varkey B., Dharmarajan T., Pare G., Bahl V.K. (2019). Lipoprotein(a): An independent, genetic, and causal factor for cardiovascular disease and acute myocardial infarction. Indian Heart J..

[10] Tokgozoglu L., Kayikcioglu M. (2021). Familial Hypercholesterolemia: Global Burden and Approaches. Curr. Cardiol. Rep..

[11] Raal F.J., Rosenson R.S., Reeskamp L.F., Hovingh G.K., Kastelein J.J., Rubba P., Ali S., Banerjee P., Chan K.-C., Gipe D.A. (2020). Evinacumab for Homozygous Familial Hypercholesterolemia. N. Engl. J. Med..

[12] Raal F.J., Santos R.D. (2012). Homozygous familial hypercholesterolemia: Current perspectives on diagnosis and treatment. Atherosclerosis.

[13] Thompson G.R. (2015). Managing homozygous familial hypercholesterolaemia from cradle to grave. Atheroscler. Suppl..

[14] Bajaj A., Cuchel M. (2020). Homozygous familial hypercholesterolemia: What treatments are on the horizon?. Curr. Opin. Lipidol..

[15] Cesaro A., Fimiani F., Gragnano F., Moscarella E., Schiavo A., Vergara A., Akioyamen L., D’erasmo L., Averna M., Arca M. (2021). New Frontiers in the Treatment of Homozygous Familial Hypercholesterolemia. Heart Fail. Clin..

[16] Kayikcioglu M., Tokgozoglu L., Yilmaz M., Kaynar L., Aktan M., Durmuş R.B., Gokce C., Temizhan A., Ozcebe O.I., Akyol T.K. (2018). A nation-wide survey of patients with homozygous familial hypercholesterolemia phenotype undergoing LDL-apheresis in Turkey (A-HIT 1 registry). Atherosclerosis.

[17] Engin A. (2017). Endothelial Dysfunction in Obesity. Adv. Exp. Med. Biol..

[18] Gamez-Mendez A.M., Vargas-Robles H., Ríos A., Escalante B. (2015). Oxidative Stress-Dependent Coronary Endothelial Dysfunction in Obese Mice. PLoS ONE.

[19] Chen M., Masaki T., Sawamura T. (2002). LOX-1, the receptor for oxidized low-density lipoprotein identified from endothelial cells: Implications in endothelial dysfunction and atherosclerosis. Pharmacol. Ther..

[20] Zhang X., Sessa W.C., Fernández-Hernando C. (2018). Endothelial Transcytosis of Lipoproteins in Atherosclerosis. Front. Cardiovasc. Med..

[21] Medina-Leyte D.J., Zepeda-García O., Domínguez-Pérez M., González-Garrido A., Villarreal-Molina T., Jacobo-Albavera L. (2021). Endothelial Dysfunction, Inflammation and Coronary Artery Disease: Potential Biomarkers and Promising Therapeutical Approaches. Int. J. Mol. Sci..

[22] Austin R.C., Lentz S.R., Werstuck G.H. (2004). Role of hyperhomocysteinemia in endothelial dysfunction and atherothrombotic disease. Cell Death Differ..

[23] Silva I.V.G., de Figueiredo R.C., Rios D.R.A. (2019). Effect of Different Classes of Antihypertensive Drugs on Endothelial Function and Inflammation. Int. J. Mol. Sci..

[24] Ron D. (2002). Translational control in the endoplasmic reticulum stress response. J. Clin. Investig..

[25] Marchini J.F., Manica A., Crestani P., Dutzmann J., Folco E.J., Weber H., Libby P., Croce K. (2020). Oxidized Low-Density Lipoprotein Induces Macrophage Production of Prothrombotic Microparticles. J. Am. Heart Assoc..

[26] Miyao M., Cicalese S., Cooper H.A., Eguchi S. (2019). Endoplasmic reticulum stress and mitochondrial biogenesis are potential therapeutic targets for abdominal aortic aneurysm. Clin. Sci..

[27] Ghosh A., Gao L., Thakur A., Siu P.M., Lai C.W.K. (2017). Role of free fatty acids in endothelial dysfunction. J. Biomed. Sci..

[28] Hong J., Kim K., Park E., Lee J., Markofski M.M., Marrelli S.P., Park Y. (2018). Exercise ameliorates endoplasmic reticulum stress-mediated vascular dysfunction in mesenteric arteries in atherosclerosis. Sci. Rep..

[29] Goulooze S.C., Cohen A.F., Rissmann R. (2015). Lomitapide. Br. J. Clin. Pharmacol..

[30] Robl J.A., Sulsky R., Sun C.-Q., Simpkins L.M., Wang T., Dickson J.K., Chen Y., Magnin D.R., Taunk P., Slusarchyk W.A. (2001). A Novel Series of Highly Potent Benzimidazole-Based Microsomal Triglyceride Transfer Protein Inhibitors. J. Med. Chem..

[31] Chacra A.P.M., Ferrari M.C., Rocha V.Z., Santos R.D. (2019). Case report: The efficacy and safety of lomitapide in a homozygous familial hypercholesterolemic child. J. Clin. Lipidol..

[32] Cefalù A.B., Giammanco A., Noto D., Spina R., Cabibi D., Barbagallo C.M., Averna M. (2020). Effectiveness and safety of lomitapide in a patient with familial chylomicronemia syndrome. Endocrine.

[33] Lupo M.G., Arcidiacono D., Zaramella A., Fimiani F., Calabrò P., Cefalù A.B., Averna M., D’Erasmo L., Arca M., De Martin S. (2021). Lomitapide does not alter PCSK9 and Lp(a) levels in homozygous familial hypercholesterolemia patients: Analysis on cytokines and lipid profile. Atheroscler. Plus.

[34] Munkhsaikhan U., Kwon Y., Sahyoun A.M., Ait-Aissa K., Kassan A., Kassan M. (2022). The Microsomal Triglyceride Transfer Protein Inhibitor, Lomitapide, Improves Vascular Function in Obesity. FASEB J..

[35] Brautbar A., Ballantyne C.M. (2011). Pharmacological strategies for lowering LDL cholesterol: Statins and beyond. Nat. Rev. Cardiol..

[36] Patel V., Joharapurkar A., Kshirsagar S., Patel M., Patel H., Savsani H., Jain M. (2021). Microsomal triglyceride transfer protein inhibitor lomitapide-induced liver toxicity is ameliorated by Triiodothyronine treatment following improved bile homeostasis and β-oxidation. Toxicol. Appl. Pharmacol..

[37] Nagy C., Einwallner E. (2018). Study of In Vivo Glucose Metabolism in High-fat Diet-fed Mice Using Oral Glucose Tolerance Test (OGTT) and Insulin Tolerance Test (ITT). J. Vis. Exp..

[38] Andrés-Manzano M.J., Andrés V., Dorado B. (2015). Oil Red O and Hematoxylin and Eosin Staining for Quantification of Atherosclerosis Burden in Mouse Aorta and Aortic Root. Methods Mol. Biol..

[39] Bourghardt J., Wilhelmson A.S.K., Alexanderson C., De Gendt K., Verhoeven G., Krettek A., Ohlsson C., Tivesten A. (2010). Androgen Receptor-Dependent and Independent Atheroprotection by Testosterone in Male Mice. Endocrinology.

[40] Zhao W.-G., Zhu H.-J. (2010). [Mechanism, treatment, and evaluation of obesity-induced insulin resistance and type 2 diabetes]. Zhongguo Yi Xue Ke Xue Yuan Xue Bao.

[41] Pathak P., Shukla P., Kanshana J.S., Jagavelu K., Sangwan N.S., Dwivedi A.K., Dikshit M. (2021). Standardized root extract of Withania somnifera and Withanolide A exert moderate vasorelaxant effect in the rat aortic rings by enhancing nitric oxide generation. J. Ethnopharmacol..

[42] Kassan M., Vikram A., Li Q., Kim Y.-R., Kumar S., Gabani M., Liu J., Jacobs J.S., Irani K. (2017). MicroRNA-204 promotes vascular endoplasmic reticulum stress and endothelial dysfunction by targeting Sirtuin1. Sci. Rep..

[43] Alonso R., Cuevas A., Mata P. (2019). Lomitapide: A review of its clinical use, efficacy, and tolerability. Core Evid..

[44] Zheng Y., Hu Y., Han Z., Yan F., Zhang S., Yang Z., Zhao F., Li L., Fan J., Wang R. (2022). Lomitapide ameliorates middle cerebral artery occlusion-induced cerebral ischemia/reperfusion injury by promoting neuronal autophagy and inhibiting microglial migration. CNS Neurosci. Ther..

[45] Pajkowski M., Chlebus K., Hellmann M. (2020). Microvascular endothelial dysfunction in a young patient with familial hypercholesterolemia. Pol. Arch. Intern. Med..

[46] Vlahos A.P., Naka K.K., Bechlioulis A., Theoharis P., Vakalis K., Moutzouri E., Miltiadous G., Michalis L.K., Siamopoulou-Mavridou A., Elisaf M. (2014). Endothelial Dysfunction, But Not Structural Atherosclerosis, Is Evident Early in Children With Heterozygous Familial Hypercholesterolemia. Pediatr. Cardiol..

[47] Kirchner H., Hofmann S.M., Fischer-Rosinský A., Hembree J., Abplanalp W., Ottaway N., Donelan E., Krishna R., Woods S.C., Müller T.D. (2012). Caloric Restriction Chronically Impairs Metabolic Programming in Mice. Diabetes.

[48] Schreyer S.A., Wilson D.L., LeBoeuf R.C. (1998). C57BL/6 mice fed high fat diets as models for diabetes-accelerated atherosclerosis. Atherosclerosis.

[49] Kameyama N., Maruyama C., Kitagawa F., Nishii K., Uenomachi K., Katayama Y., Koga H., Chikamoto N., Kuwata Y., Torigoe J. (2019). Dietary Intake during 56 Weeks of a Low-Fat Diet for Lomitapide Treatment in Japanese Patients with Homozygous Familial Hypercholesterolemia. J. Atheroscler. Thromb..

[50] Dhote V., Joharapurkar A., Kshirsagar S., Dhanesha N., Patel V., Patel A., Raval S., Jain M. (2011). Inhibition of microsomal triglyceride transfer protein improves insulin sensitivity and reduces atherogenic risk in Zucker fatty rats. Clin. Exp. Pharmacol. Physiol..

[51] Phillips C., Owens D., Collins P., Tomkin G. (2002). Microsomal triglyceride transfer protein: Does insulin resistance play a role in the regulation of chylomicron assembly?. Atherosclerosis.

[52] Church T., Martin C.K. (2018). The Obesity Epidemic: A Consequence of Reduced Energy Expenditure and the Uncoupling of Energy Intake?. Obesity.

[53] McAllister E.J., Dhurandhar N.V., Keith S.W., Aronne L.J., Barger J., Baskin M., Allison D.B. (2009). Ten putative contributors to the obesity epidemic. Crit. Rev. Food Sci. Nutr..

[54] Rosei E.A., Salvetti M. (2016). Management of Hypercholesterolemia, Appropriateness of Therapeutic Approaches and New Drugs in Patients with High Cardiovascular Risk. High Blood Press. Cardiovasc. Prev..

[55] Lairon D., Lopez-Miranda J., Williams C. (2007). Methodology for studying postprandial lipid metabolism. Eur. J. Clin. Nutr..

[56] Ueshima K., Akihisa-Umeno H., Nagayoshi A., Takakura S., Matsuo M., Mutoh S. (2005). Implitapide, a Microsomal Triglyceride Transfer Protein Inhibitor, Reduces Progression of Atherosclerosis in Apolipoprotein E Knockout Mice Fed a Western-Type Diet: Involvement of the Inhibition of Postprandial Triglyceride Elevation. Biol. Pharm. Bull..

[57] Vakkilainen J., Mäkimattila S., Seppälä-Lindroos A., Vehkavaara S., Lahdenperä S., Groop P.-H., Taskinen M.-R., Yki-Järvinen H. (2000). Endothelial Dysfunction in Men With Small LDL Particles. Circulation.

[58] Gimbrone M.A., García-Cardeña G. (2016). Endothelial Cell Dysfunction and the Pathobiology of Atherosclerosis. Circ. Res..

