# Supplementary material for: The Beneficial Effect of Lomitapide on the Cardiovascular System in LDLr−/− Mice with Obesity
Source: Antioxidants (Basel). 2023 Jun 16;12(6):1287. doi: 10.3390/antiox12061287 (PMC10295391; doi:10.3390/antiox12061287)
Supplement: Supplementary file 1 [file antioxidants-12-01287-s001.zip › antioxidants-2289934-supplementary.pdf]

Supplementary Table 1. RT-qPCR primers are used for aorta gene expression analysis.

**BIP**	TGTCTTCTCAGCATCAAGCAAGG	CCAACACTTCCTGGACAGGCTT
**ATF6**	GTCCAAAGCGAAGAGCTGTCTG	AGAGATGCCTCCTCTGATTGGC
**ATF4**	AACCTCATGGGTTCTCCAGCGA	CTCCAACATCCAATCTGTCCCG
**NOX2**	TGGCGATCTCAGCAAAAGGTGG	GTACTGTCCCACCTCCATCTTG
**P65**	TCCTGTTCGAGTCTCCATGCAG	GGTCTCATAGGTCCTTTTGCGC
**P50**	GCTGCCAAAGAAGGACACGACA	GGCAGGCTATTGCTCATCACAG
**TNFα**	GGTGCCTATGTCTCAGCCTCTT	GCCATAGAACTGATGAGAGGGAG
**VCAM1**	GCTATGAGGATGGAAGACTCTGG	ACTTGTGCAGCCACCTGAGATC
**GAPDH**	CATCACTGCCACCCAGAAGACTG	ATGCCAGTGAGCTTCCCGTTCAG
